# REDLARA: greater role than that of a technological society

**DOI:** 10.5935/1518-0557.20140017

**Published:** 2014

**Authors:** Maria do Carmo Borges de Souza

**Affiliations:** REDLARA President

Most Latin American countries have adopted the WHO Programme, which brings a comprehensive concept of reproductive health (Beijing, UN, 1995): women are entitled to the highest standards of physical and mental health and the exercise of this right is vital to their welfare conditions and to actively participate in all areas of their public and private life.

In Latin America, inequality or inequity is a common condition and affects men and women, but woman is the weakest link in regard to the formation of a family. Health and Wellness do not include most Latin American women with dependent determinants of geographical, social, political and economic context, beyond the biological.

Ever wondered if our geography would equal just “destiny”, since geographical conditions affect the development in several ways; or mean determinism, since in Latin America there are income levels and health conditions equal to typical regions of Africa, along with places where income, health and education are much closer to the best standards of the industrialized world.

We must look ahead and see LA’s multiculturalism and geography also determining strong ties within this context. These ties, for example, led scientifically to REDLARA, whose successful existence again joins us in the current debate and intense search of the best ways to exchange experiences and promote a set of possible solutions, which may also result social improvement of our people in different countries.

Human reproduction is part of “health promotion” process, with options that should be based on education and expansion of individual and collective rights, leading to a better living. In general what we are seeing is the maturity of our societies in LA: there is a search for general principles of law (ART techniques necessary and effective for the purpose of procreation and not contrary to any country’s Constitution, laws or rights of third parties. Ethical-legal reasonable proposals are being discussed in order to achieve common denominators between different existing points of view in order to reach a reasonable and sustainable agreement, clear definition of the ART professionals and centers that can perform them, with rules controlled by the health authority.

Any draft legislation should, however, seek to consecrate broadly the right people has to get to ART without economic discrimination as another barrier. In this REDLARA’s Border of Directors and specialists are being consulted throughout Latin America. Hands on, everybody.

**Maria do Carmo Borges de Souza**

REDLARA President

